# Elite female Gaelic sports athletes’ experience of urinary incontinence: A qualitative study

**DOI:** 10.1177/17455057251406949

**Published:** 2025-12-24

**Authors:** Elizabeth Culleton-Quinn, Kari Bø, Neil Fleming, Cinny Cusack, Déirdre Daly

**Affiliations:** 1 School of Medicine, Trinity College, Dublin, Ireland; 2 Norwegian School of Sports Sciences, Oslo, Norway; 3 Department of Obstetrics and Gynecology, Akershus University Hospital, Lørenskog, Norway; 4 Physiotherapy Department, Rotunda Hospital, Dublin, Ireland; 5 School of Nursing and Midwifery, Trinity College, Dublin, Ireland

**Keywords:** urinary incontinence, experience, elite female athletes

## Abstract

**Background:**

Urinary incontinence (UI) is known to be prevalent among elite female athletes. A need for additional sports-specific research has been identified, along with a need to understand female athletes’ experience of UI. There is a dearth of research on elite Female Gaelic (Irish) sports players competing in national competitions.

**Aim:**

To investigate elite female Gaelic sports (Camogie and Football players) athletes’ experiences of UI.

**Design:**

A qualitative descriptive design using one-to-one interviews.

**Methods:**

Following University ethics approval, purposive sampling was used to recruit elite female Gaelic sports athletes aged 18 years or older playing at the county level between November 2022 and August 2023. Data were analysed thematically using reflexive thematic analysis, a six-phase process.

**Findings:**

Nine players consented to and completed an interview. Four key themes and nine sub-themes were generated. Four themes were: (1)‘Oh, that happened me’ (when and why of UI); (2) ‘Then I can manage it that way’ (what to do about UI); (3) ‘It is frustrating. . . and it bothers me’ (impact of UI) and (4) ‘No one ever talked’ (talking about UI). High-intensity and high-impact activities were identified by all players as triggers for UI, suggesting stress urinary incontinence. Strategies to manage UI were used by all nine players and included extra and frequent pre-match voiding, wearing protection, and modifying their clothing or fluid intake. Players minimised the extent of their UI; however, all spoke of experiencing either a physical or an emotional impact because of the UI.

**Conclusions:**

This study provided qualitative evidence on elite female athletes’ experience of UI. Players used strategies to self-manage their UI, few spoke about, or sought help for their UI whilst many expressed a desire for education. Findings suggest a need for education programmes among players to include information about the condition of UI as well as available treatment options.

## Introduction

Urinary incontinence (UI) is the most commonly experienced pelvic floor disorder (PFD) in women and has been defined as the ‘complaint of involuntary loss of urine.’^1^ The prevalence of UI has been found to be higher among female athletes compared to their non-athletic counterparts. Recent systematic reviews have also indicated that a higher prevalence of UI has been found to be associated with high-impact sport, competition at an elite level, and long hours of training.^2–12^ Moore et al. highlighted the fact that a supplement to the International Olympic Committee 2020 consensus statement, regarding epidemiological data on illness and injury in sport, had a focus on the female athlete. Of the 10 female health domains identified, one related to pelvic floor health, including UI. Examples were given as to how urinary symptoms may potentially affect participation and performance of the female athlete and included female athletes reducing their training due to embarrassment and fear of experiencing UI, as well as urinary frequency disrupting their participation.^13^

Much of the qualitative research to date regarding the experience and impact of PFD has focused on women with UI,^14,15^ on women during pregnancy or following childbirth,^16,17^ following gynaecological cancer surgery^18^ or older adults.^19^ A meta-ethnography reviewing 41 primary qualitative studies explored the experience of men and women with UI found that a culture of secrecy and feelings of shame acted as a barrier to seeking help for UI.^20^ The experience of UI has been shown to have a marked impact on women, and there is a need for additional studies to investigate the impact of UI on the quality of life of younger women.^21^ A mixed-methods systematic review by Dakic et al. investigated the effect that symptoms of PFD had on women’s participation in exercise, with 50% of women with UI reporting that they either modified or stopped exercise as a result of their symptoms.^22^

There is limited research to date exploring the experience of PFD among elite female athletes.^23^ A qualitative study by Dakic et al. explored the experience of women within sports and exercise settings and included both recreational and elite sportswomen. Pelvic floor symptoms impacted on the women’s participation, affecting choice, frequency and intensity levels of exercise. In addition, the women reported experiencing negative emotions as a consequence of their UI. Women used coping strategies to manage their UI, but these impacted negatively on the social benefits and spontaneity of participating in exercise. The authors identified a need for increased awareness of PFD symptoms among sports and exercise organisations and a need for education and screening of symptoms of PFD.^24^

Only one study to date has involved a qualitative component when investigating the impact of UI focusing on elite female athletes. A mixed-methods study by Jácome et al. incorporated a small focus group (*n* = 7) with athletes reporting strategies to manage UI, emotional responses of frustration, annoyance and fear concerning their experience of UI. None of the athletes sought treatment for their UI. The authors recommended a need for increased awareness regarding UI in sport and research to further investigate the experience of athletes with UI.^25^

Gaelic team sports are the national sports in Ireland, and the female sports include Camogie and Ladies Gaelic Football and both are sports involving two 15-member teams playing on a large grass pitch and players and involve high-impact activities such as jumping, catching and landing as well as running and sprinting. A hurley (stick) and a sliotar (small, hard, leather-covered ball) are used in the game of Camogie and players strike the sliotar with the hurley, solo (move with the sliotar bouncing or balanced on the hurley), handpass or kick the sliotar.^26^ A round football is used in Ladies Gaelic football with players kicking, catching, soloing (players drop the ball onto their foot and kick it back into their hands), bouncing or handpassing the ball.^27^ Camogie and Ladies Gaelic Football players playing for county teams and competing in National championships represent elite players in these two sports.

The study presented here is the qualitative component of a mixed-methods study investigating the prevalence and experience of UI elite female Gaelic sports athletes.^28^ This qualitative study sought to address a gap in the research to date exploring UI among elite female athletes and may help researchers and those involved with elite female Gaelic sports athletes gain a better understanding of the lived experience of UI from the players’ perspective. New knowledge may emerge and could potentially inform policy and practice in this area. Therefore, the aim of this study was to investigate the experience of UI among elite female Gaelic sports athletes.

## Methods

### Study design

This research utilised a qualitative descriptive study design^29^ and was underpinned by pragmatism as a philosophical framework.^30^ Reasons for choosing this framework include the fact that, whilst some philosophical approaches, such as positivism, focus on the nature of reality, pragmatism focuses on the nature of experience.^31^ The COnsolidated criteria for REporting Qualitative research (COREQ) reporting guidelines^32^ were followed, and the COREQ checklist was completed (Supplemental Appendix 1).

### Sampling, selection criteria

Participants were selected using purposive sampling and were elite female Gaelic sports athletes aged 18 years of age or older, who played at the County level within the last 5 years, able to understand written and spoken English and had a self-identified history of experiencing UI. Players who had previous pelvic floor surgery or who had given birth within the previous 12 months were excluded.

### Recruitment and data collection

Following university ethical approval, the Ladies Gaelic Football and Camogie associations, the official representative bodies of the two sports, acted as gatekeepers for the study. The associations sent out study information by email to County players in November 2022 and posted information on their social media outlets on a monthly basis from November 2022 until August 2023, when the study closed. Players interested in participating contacted the principal investigator (PI) by email and were sent a study pack including a participant information leaflet and consent form. One-to-one interviews were conducted online using Microsoft Teams and transcribed using Microsoft Streams.

A semi-structured interview guide (Supplemental Appendix 2) was used and was informed by findings from an earlier quantitative study^28^ and by consultation with a number of experts, including former female Gaelic sports athletes (*n* = 4), physiotherapists involved in treating field sports athletes (*n* = 2) and pelvic health physiotherapists (*n* = 3).

### Data analysis

Interview transcripts were transferred verbatim, checked for accuracy and entered line by line into an Excel spreadsheet. The data were coded manually and thematically analysed using the six phases of reflexive thematic analysis (RTA) as outlined by Braun and Clarke. This involved the researchers being both reflective and thoughtful when engaging with the data as well as with the research process.^33^ Two members of the research team (ECQ and DD) conducted the analysis.

### Trustworthiness and rigour

Four criteria to establish overall trustworthiness of the research process were identified by Lincoln and Guba as credibility, transferability, dependability and confirmability^34^ and steps were taken throughout this study to facilitate trustworthiness and rigour. The PI used peer debriefing as well as a reflective diary throughout this study to maintain a decision and audit trail.^35^ All transcripts were cross-checked twice to confirm accuracy. Member checking^36^ was conducted, initially by returning the interview transcripts to the participants and, subsequently, by sending a synopsis of the key findings, including themes, sub-themes and the thematic map, so that the participants could check if their views were reflected in the findings.

### Ethical considerations

Ethical approval was obtained from the School of Nursing and Midwifery Ethics Committee at Trinity College, Dublin (Reference: SMNREC COM_13.20).

### Consent to participate

Participants completed signed, written consent prior to the interview and verbally confirmed consent at the start of the interview.

## Findings

Fifteen players expressed an interest and were sent the study pack. Five players did not respond, and one player responded but withdrew before the interview. Subsequently, nine, one-to-one online interviews were conducted between December 2022 and June 2023. The background characteristics of the nine players are shown in Table 1. The mean age (standard deviation (SD)) of the players was 28.2 (4.5) years. The interview duration ranged from 15.3 to 27.5 min (mean (SD) = 20.6 (3.8) min).

**Table 1.** Players’ characteristics.

Player	Age (years)	Parity	Sport
P1	33	Nulliparous	LGF
P2	29	Nulliparous	C&LGF
P3	19	Nulliparous	C&LGF
P4	23	Nulliparous	C
P5	27	Nulliparous	LGF
P6	31	Nulliparous	LGF
P7	30	Nulliparous	C
P8	30	Nulliparous	LGF
P9	32	Parous	LGF

C: County Camogie Player; LGF: County Ladies Gaelic Football Player.

### Themes, sub-themes and data saturation

Following RTA, four key themes and nine sub-themes regarding the players’ experience of UI were generated. The four themes were: (1) ‘Oh, that happened me’ (the when and why of UI); (2) ‘Then I can manage it that way’ (what to do about UI); (3) ‘It is frustrating. . . and it bothers me’ (impact of UI) and (4) ‘No one ever talked’ (talking about UI). Table 2 displays the four themes and associated sub-themes.

**Table 2.** Players’ experience of UI: themes and sub-themes.

Themes	Sub-themes
1. ‘Oh, that happened me’ (the when and why of UI)	1.1. When UI happened 1.2. Triggers for UI
2. ‘Then I can manage it that way’ (what to do about UI)	2.1. Strategies to manage UI 2.2. The need for education (about UI and treatment options)
3. ‘It is frustrating. . . and it bothers me’ (impact of UI)	3.1. Impact of UI: physical and practical concerns 3.2. Impact of UI: emotional impact
4. ‘No one ever talked’ (talking about UI)	4.1. Reticent to disclose UI 4.2. Normalising/minimising UI 4.3. Talking about UI with others

UI: urinary incontinence.

Hennink et al. identified that a small number of interviews may sufficiently capture a range of issues and themes generated from the data. Subsequently, more data may be needed to further develop and enrich the understanding of the themes and sub-themes generated from the data.^37^ The range of themes and sub-themes outlined in this study was identified by the sixth interview. The final three interviews helped enrich the understanding of the themes and sub-themes that had been generated, and data saturation was established.

### Theme 1: ‘oh, that happened me’ (the when and why of UI)

This theme describes the players’ experience of UI with regard to times when they noticed UI was happening, as well as triggers for their UI.

#### Sub-theme 1.1: when UI happened

The timing of when players experienced UI during sport varied, with some reporting UI at the warm-up or start of matches or training and spoke of how their UI was linked with particular movements, such as high-impact movements, suggesting stress urinary incontinence (SUI).

‘Erm probably matches. I’d noticed it [UI] bit more during the warm-up because I’d be. . . jumping. . .’ (P2, C&LGF)

‘. . .especially at the start of training and with the jumping and the bounding. . .I would leak then.’ (P9, LGF)

Others associated their UI with fatigue at the end of training or matches. Players described experiencing UI towards the end of more strenuous and ‘bigger’ matches, associating their UI with tiredness, fatigue and more tackling.

‘Yeah. like you would notice [UI] particularly in, like, bigger games that were much more physically draining. . . I wouldn’t have experienced it really in challenge games or lighter games. . .it was always just those really draining, exhausting games. . .the more tired I got then I noticed it [UI].’ (P6, LGF)

‘I would say [pause] probably more so towards the end of a match. . . where I’ve had no leakage. . . always tend to be those easier games. . .I don’t take as many tackles. . .But definitely would notice [UI], especially say once you’re hitting like semi-finals/finals of championships. . . I’m. . .pre-prepared for leaking in those games.’ (P7, C)

Players spoke of experiencing urinary urgency and urinary frequency just before the start of a match, particularly a big game and they were nervous.

‘. . .if I’m nervous or like before a match then kind of nerves are kicking in. . .I know I’ll have gone to the toilet, but I’ll just feel that sudden urge to go again.’ (P2, C&LGF)

Some players said they were more aware of their UI at certain times in their menstrual cycle.

‘Well, my cycle will affect it [UI] definitely. . .just prior to me getting my erm period it [UI] will be more significant for me.’ (P7, C)

Many players said that it was during their teenage years when they first noticed UI during sports.

‘I would say I’ve been playing football over 16 years, but I’d say I first started noticing it [UI] maybe when I was like 16 or 17 (years).’ (P5, LGF)

#### Sub-theme 1.2: triggers for UI

A wide variety of triggers for UI were reported by the players. All players mentioned that more strenuous, high-intensity activities were triggers for their UI.

‘. . .It’s [UI] only when I push myself to like my max. . . yeah more it’s like when you’ve really hit your full point of exertion. . .’ (P1, LGF)

‘Yeah, I would have noticed it [UI] kind of in high intensity training or running.’ (P8, LGF)

Certain movements and activities, such as high-impact activities including jumping and landing, were triggers for UI, suggesting that symptoms of SUI were commonly experienced by the players.

‘. . .or there’s any kind of jump involved, it’s normally around then I notice it [UI] a lot.’ (P2, C&LGF)

Physical contact during games, such as tackles, kicking a ball or striking the sliotar, was trigger for UI.

‘. . .So, if I’m in a short strike pass, it’s usually fine. . .but if I’m really obviously trying to put that kind of bit of an umph behind it, yeah, definitely. And especially after. . .I’ve had to break a tackle. . . it’s [UI] more likely to happen.’ (P7, C)

Both the amount and type of fluid that players drank impacted their UI and urinary frequency.

‘. . . if I’ve drank too much you know then it would be like just you might leak during a game. . .because of the amount of liquid in you. . . one thing I suppose is the sports drinks you know would have to like they definitely flow through me quicker. . .I could leak then easier. . .’ (P1, LGF)

‘. . .I go nearly every hour, every two hours. And I drink a lot of water, which probably contributes to that [urinary frequency] as well.’ (P5, LGF)

### Theme 2: ‘then I can manage it that way’ (what to do about UI)

Players spoke about how they managed their UI, and many expressed a desire for further knowledge.

#### Sub-theme 2.1: strategies adopted by players to manage UI

All nine players used a minimum of two strategies to manage their UI during their sporting activity. Strategies included extra voiding, wearing protection, modifying clothing, modifying fluid intake, seeking treatment and carrying out PFMT. Some of the players used as many as four strategies.

Extra voiding was used by most players. Although it is not an uncommon practice to pass urine before competition, players felt that needing to go a number of times in quick succession before a match was a distraction. In addition, they spoke of the need to void frequently at training and at matches and control their hydration to ensure that they had an empty bladder to minimise leaks. Players spoke of voiding at halftime, with the extra voiding linked with a fear of UI rather than a need to void.

‘. . .oh, like I’d always go like to the toilet right before we go out for the first time. . .half time as well, even though I never needed to actually go really at half time. . .if there was a queue for the toilet, I’d still have to wait and wait. . .I might be the last person on the pitch. . .like you just want to be able to focus on the performance.’ (P1, LGF)

Some form of protection was worn by most players as a strategy to manage UI, ranging from pantiliners, period knickers to pads. Players spoke of how wearing protection was not always effective or comfortable, and some used pantiliners as a precaution in case of a leak.

‘Erm I actually use pantyliners most of the time when I’m playing just in case.’ (P2, C&LGF)

‘. . .I usually wear a. . . continence pad. . . don’t feel overly confident at using some of the internal devices. . . I’m actually afraid it’s going to come out. . .in the middle of a game. . .that’s not going to be something I can manage [laugh]. And so. . .usually I would wear a continence pad.’ (P7, C)

‘I definitely feel maybe a pad, or something might [help], but. . .I find they’re not that comfortable. . .’ (P1, LGF)

Needing increased protection following childbirth was mentioned, with varying sizes of pads and period knickers required for added protection.

‘So, for me, especially after having my baby I definitely would have had to wear a pad for training all the time. . . a heavier pad on if I needed to. . .I also got those period knickers. . .If I was playing a match now like it would be rare if I’m not wearing the period knickers. . .’ (P9, LGF)

Players spoke about modifying or changing their clothing for comfort and to avoid any leakage being seen on their outer clothing. Modifications included wearing a second layer of clothing, such as skin-tight shorts under their sports shorts, or wearing dark shorts to prevent leaks showing.

‘. . .I would have worn. . .under shorts. . . skin-tight ones. . .more support and also protection. . .And I would wear black ones even though we had white shorts. . . because even if I did [leak] the chances of that then leaking through to your shorts were less likely. . .’ (P6, LGF)

Players recognised the importance of adequate hydration and, even though the amount and type of fluid could act as a trigger for UI, many said that limiting their fluid intake was not a strategy that they utilised.

‘If anything, I drink, I drink more water coming up to big games like.’ (P3, C&LGF)

‘I don’t [restrict fluids]. Like I just maybe that’s potentially why it [UI] happens in those bigger games, because. . . I’m obviously more conscious of making sure that my fluid intake has been good. . .’ (P7, C)

However, some players spoke of limiting their fluids as well as timing their fluid intake to manage their UI.

‘. . .I have to then be aware. . . you know, an hour and a half before kick-off I can’t really drink that much more. . .that’s when. . .you shouldn’t be limiting yourself. . . I suppose it [UI] is never huge once I kind of managed it. . . with. . .not drinking that much . . .’ (P1, LGF)

Most players had not sought treatment from a healthcare professional to manage their urinary symptoms. One received help from her GP for nocturia, and two sought help from a pelvic health physiotherapist.

‘. . .when I was in college, I was on tablets for incontinence because of getting up in the middle of night. . .But that was probably the only time, I’ve ever spoken to them [HP] about my bladder.’ (P5, LGF)

‘I went to a pelvic floor specialist after I had [baby’s name]. That was kind of been more in relation to like. . .was it safe for me to go back playing and stuff?’ (P9, LGF)

Three players spoke of the role of the pelvic floor and used PFMT as a strategy to manage their UI. Two of these players worked in healthcare and one player had given birth.

‘. . .when I am more consistent with my pelvic floor exercises, it [UI] is a lot less and. . .if I haven’t been very good with my pelvic floor exercises, I. . .notice it [UI] more.’ (P7, C)

‘Yeah, I probably am not doing it specifically but like I’d be in the gym a good bit. So, you are kind of doing it [PFMT] then anyway.’ (P9, LGF)

#### Sub-theme 2.2: need for education regarding UI and treatment available

Many of the players had limited knowledge about UI and PFMT with many expressing a desire for education regarding UI and treatment options. Players were also unsure who to approach to ask for information or treatment.

‘. . .I’ve never sought that advice. So, if. . . you injured your ankle and you go to a physio. But. . .there’s not that for this this [UI]. I’m having a bladder issue. Who do I go to talk to? The GP? They’re just going to tell you to do Kegels I don’t even know if I’m doing it right.’ (P5 LGF)

Many players spoke of obtaining information from the internet and from friends and family members, and spoke of not being confident in their knowledge of how to carry PFMT effectively.

‘I like Googled it a lot. . .it was saying kind of that [pause]. . . Well, no, I wouldn’t be confident. . .that I was doing them right. . .’ (P1, LGF)

‘Well muscles in your pelvic floor and you can strengthen them. . .I wouldn’t know how to start [PFMT]. I know how to do exercises on my legs or my arms or my core, but I don’t know [about PFMT].’ (P3 C&LGF)

Players expressed a desire for education regarding UI, with suggestions including education within schools or podcasts for sportswomen similar to ones available to postnatal women.

‘. . .if maybe it was brought up in some kind of team thing. . .Or maybe if you saw like Ads. . .should we be learning about that in school?. . . or at least knowing that maybe this is the thing we could be doing, you know? And. . .bring it into, female sports. . .’ (P1, LGF)

### Theme 3: ‘it is frustrating. . . and it bothers me’ (impact of UI)

Players spoke of the physical and emotional impact of UI and of their practical concerns in response to their experience of UI.

#### Sub-theme 3.1: impact of UI – physical and practical concerns

Practical concerns included issues such as personal comfort as well as those associated with the availability of facilities. Players spoke of having to deal with feeling damp due to their UI, training in damp clothing and unable to change.

‘. . .I had to play the rest of the training, and I could feel that like I was damp. . .probably should have been more prepared for it. . .’ (P5, LGF)

Many players also spoke of how it was not often possible to access toilets or changing facilities and how the cleanliness of facilities impacted their experience.

‘It’s very hard to train or even to play if you need to go to the bathroom. And sometimes the toilets wouldn’t be open at pitches, especially in the GAA. . .there’s only men they take into consideration [laugh]. . .. And. . .sometimes there would be a leak or something. . ..’ (P3, C&LGF)

‘Definitely not no! . . .we train on like the pitch that’s like three pitches away from the toilets. . .I’d be in the middle of training, and I have to go in the ditch. . .if we had a Portaloo there would be like a little bit better. . .a few others just go to the toilet in behind the shed [laugh].’ (P5, LGF)

#### Sub-theme 3.2: impact of UI – emotional impact on players

All players expressed at least one emotional response because of their experience of UI during sport. Players spoke of being embarrassed and self-conscious in case there was an odour. In addition, players were uncomfortable to disclose their UI when their coach was male.

‘. . .like a lot of girls and women’s teams have many men coaches and if it’s [UI] not happening to both men and women, it’s just not something talked about as much because. . .they might feel not comfortable talking about it.’ (P2, C&LGF)

‘Say, if I was to give somebody a lift. . .after a match or training, then I’m kind of thinking, ‘Oh, God, like . . .can they smell’ . . .like, you know [leak]. . .I am a little bit self-conscious about it.’ (P4, C)

Many players expressed how they felt bothered, irritated, annoyed, or frustrated by their UI.

‘. . .. my mom. . .just says it’s [UI] normal that it’s going to happen, but it is just really annoying.’ (P2, C&LGF)

‘It’s just irritating. . .I trained that whole training session with damp shorts and knickers. . .I didn’t have anything with me because I don’t know I didn’t expect it [UI]. So, it is like frustrating. . .and it bothers me.’ (P5, LGF)

Players were concerned that a leak could show on their shorts or worried their pads might slip off potentially distracting them from their performance.

‘. . .a pad. . .. You know. . .it’s just something else to worry about.’ (P1, LGF)

Some players used emotive terms such as nightmare or terrible when describing their experience, and others expressed shock or surprise, shock or helplessness.

‘I played a match once. . .we were after travelling nearly two hours to get there and I was expecting a toilet and there was no toilet. . .I played horrifically. . .half time someone arrived to open the bathrooms, but it was it was a nightmare. [laugh].’ (P3, C&LGF)

‘. . .like when it first started happening, I’d kind of be like, “Oh, my God, what is this?”’ (P2, C&LGF)

### Theme 4: ‘it’s fine like yeah, no one ever never even talked’ (talking about UI)

Players spoke of the silence around the experience of UI and felt that experiencing UI was just a part of their sporting activity, and often minimised how much it impacted them.

#### Sub-theme 4.1: reticent to disclose UI

Some players were initially reticent to disclose the extent of their experience with UI. Many players initially said that they did not experience UI, but then immediately disclosed when they did in fact experience UI giving details.

‘I can’t say I have experience of it [UI] in sport but [pause] or maybe. . .I do actually. . . I’m starting I’m trying to think now. . .I’d say landing after a jump landing. . .would be probably the most common time.’ (P1, LGF)

‘Erm no, it wouldn’t affect my sport unless [pause]. . . if you needed to go to the bathroom and you were trying to hold it in, it obviously. . .it would affect you.’ (P3, C&LGF)

#### Sub-theme 4.2: normalising and minimising UI

Players often minimised their experience of UI, and many felt it may be normal for players to experience UI during sport.

‘. . .sometimes there would be at leak or something, but it wouldn’t be anything’s too serious. Yeah [laugh].’ (P3, C&LGF)

‘It [UI] was just like a thing like everything else. I mean, yeah, it was just something that was. . .a part of playing.’ (P6, LGF)

‘. . .I’d grown up with my mother being like, you know, this is just a normal thing. . .when she goes running like she always leaks. . .So, I grew up thinking it [UI] was kind of normal, so that’s why I never really looked for help.’ (P9, LGF)

#### Sub-theme 4.3: talking with others about UI

Many players spoke of the fact that UI was not discussed within the team, whereas there would be conversations about menstruation and other issues.

‘No, not at all. . . I think, players probably would talk about periods and stuff, but. . . I’ve never heard anyone speak of wee [laugh].’ (P3, C&LGF)

‘Erm no, never did, no at any point. . .Not a thing and would never have mentioned it at any point to anybody in and not out of kind of being embarrassed by it if just seemed that it was [pause] hand in hand with football and that it was part of it.’ (P6, LGF)

Some players talked about their UI with team members and said having a player who had given birth or a healthcare professional on the team could facilitate some discussion about UI.

‘. . .quite a few of us would chat about it. . . possibly because I’m a [health professional]. . .probably am not actually as open about myself, but I would talk a lot about urinary incontinence. . .a few of our girls who would talk about it, but very much so only the girls that have had babies. . .I think that’s because they feel like it’s OK for them now to have it. Whereas I’m not really sure if beforehand if they would have spoken about it.’ (P7, C)

Players spoke of how they talked with family members or friends rather than teammates whereas players with a background in healthcare were more likely to talk to a healthcare professional about their UI.

‘. . .No, just at home, to be honest, just with my own mam. . .it [UI] doesn’t happen to my sister. . .so it’s just me. . .That’s all I’ve discussed with anybody.’ (P4, C)

‘But I feel more comfortable saying it [about UI] to my family and close friends than I would be to the girls on the team.’ (P5, LGF)

‘I. . .touched base, with the pelvic health physios [pause]. . .just to get better tips to make sure that I was doing it [PFMT] right. . .I don’t think if I wasn’t a [healthcare professional] myself, I probably wouldn’t have spoken to a pelvic health physio about it. . .’ (P8, LGF)

## Discussion

Much of the findings to date regarding the experience of elite female athletes with UI have been derived from quantitative data^23^ with only one mixed-methods study involving a focus group.^25^ This current qualitative study provides information regarding elite athletes’ lived experience of UI during their sporting activity.

Players spoke of when their UI happens and identified triggers for their UI. Many players spoke of high-impact activities and high-intensity activities such as jumping and landing, and sprinting, as well as physical contact activities such as striking a sliotar or kicking a football or during tackling, as triggers for their UI. This suggests that SUI, defined as the ‘complaint of involuntary loss of urine on effort or physical exertion (e.g. sporting activities), or on coughing or sneezing’^1^ was experienced by the players. Similarly, research to date has indicated that SUI is the most common form of UI experienced by female high-impact sports athletes and is associated with an increase in abdominal pressure and ground reaction forces impacting the pelvic floor musculature.^2,6,7,9,12,38–40^ Urinary urgency and urge urinary incontinence (UUI) were also experienced and players associated urgency with when nervous just before the start of a match and went on to identify the importance of having toilet facilities available. Whilst SUI has been shown to be the most commonly experienced type, research has indicated that many female athletes also commonly experience other forms of urinary symptoms and UI.^2,4,23,28,41^ There appears to be limited research to date regarding the causation and associated factors of UUI among female athletes.^39^

Some players spoke of experiencing UI towards the end of training and matches, and in particular, the end of more strenuous matches, suggesting that pelvic floor muscle fatigue may have contributed to experiencing UI. Previous research has also indicated that female athletes are more likely to report UI towards the middle or end of competition and or training^38^ and that fatigue of the PFMs^6,42^ or long hours of training^40^ may predispose athletes to UI. A cross-sectional study investigating UI among elite Gaelic sports players found that engaging in longer hours of sporting activity per week was a risk factor for UI, again suggesting that PFM fatigue may be a factor for UI among the players.^28^

There is limited research investigating the influence of the menstrual cycle on UI, with a suggestion that hormonal variations during the cycle may lead to an increase in UI just prior to, or during menstruation^43^ but that additional research is warranted.^44^ Interestingly, some players in this study noticed an association between their menstrual cycle and UI and also spoke of noticing an increase in UI just prior to or during their period. Future research investigating the prevalence and experience of UI among female athletes should include an enquiry about UI and the menstrual cycle. Many players first noticed UI when playing sport during their teenage years. A systematic review reported an overall prevalence of 48.8% among adolescent female athletes with increased prevalence among impact sports players.^10^

A systematic review investigating the experience of UI among elite female athletes identified that elite athletes commonly use strategies to manage the symptoms of UI, whilst few sought treatment.^23^ A recent cross-sectional study found that the majority of elite Gaelic sports athletes used strategies to manage their UI, with extra voiding and wearing protection being the most commonly used strategies. Other strategies included changing or modifying clothing and reducing fluid intake.^28^ In this current study, all players used at least two different strategies to manage their UI and, similarly, extra voiding and wearing protection were the most frequently used. Players also spoke of modifying their clothing and fluid intake. The players spoke in greater detail and gave context to the use of strategies. Some players spoke of being distracted by extra voiding in quick succession before a game and voiding out of a fear of leaking rather than a need to void. The types of protection used by players varied from pantiliners worn ‘just in case’ to continence pads and period knickers. However, players did not always find wearing protection sufficient protection against leaks, nor did they always feel it was comfortable. Some players modified their clothing by wearing tight undershorts, giving added protection. A recent cross-sectional study investigating UI among female athletes participating in a Commonwealth Games reported that wearing tight-fitting sportswear meant that pads could not always be used for protection. The authors highlighted the importance of comfortable clothing for female athletes experiencing UI.^39^ Many players in this current study spoke of a preference for dark shorts or undershorts to hide any leaks. The Ladies’ Gaelic football players wear shorts during competition. At present, due to its association’s rules, Camogie players during competition must wear a skort (a pair of shorts resembling a skirt). However, most players prefer shorts as many report that a skort is uncomfortable and distracting, and that there is no freedom or flexibility when wearing them. The rule is still in place, but campaigns have been run in an attempt to have the rule removed.^45,46^ The Camogie players in this study were not specifically asked if wearing a skort had any bearing on their experience of UI, but this may be a question for future research. A systematic review has identified that pre-exercise hypohydration may impair exercise performance.^47^ Whilst players in this current study spoke about the importance of adequate hydration, some players did use fluid restriction as a strategy to manage their UI, which may be of concern.

All players spoke of how their UI evoked emotional responses such as embarrassment, frustration, annoyance, feeling self-conscious or had practical concerns surrounding their personal comfort. Such findings reflect those outlined in earlier and predominantly quantitative research among elite athletes.^23^ This current study helped contextualise and deepen the understanding of the impact of UI on the players. The importance of clean changing and toilet facilities was highlighted and could be addressed easily. Some players were concerned that their UI could be noticed by others, which reflects the findings of the qualitative component of the mixed-methods study by Jacome et al.^25^

Despite the emotional impact that UI had on players, many ‘normalised and minimised’ their UI and spoke of believing that it was normal to experience UI during sport. UI was not usually discussed within the team, and players often spoke to family and friends rather than their teammates, coaches or health professionals. At times, the advice received from family members also reinforced players’ perception that experiencing UI may be normal when exercising. Research has indicated that the level of awareness and knowledge regarding the condition of UI and treatment options available is low.^48^ For some players, the study interview was the first time that they had spoken of their UI and others were initially slow in disclosing the extent of their UI, suggesting that UI among players may be underreported. This is similar to other studies that reported a reticence among elite female athletes to disclose their UI.^2,6,39^ One study by Dos Santos that aimed to quantify urine loss with a modified pad test found that 24% of those who did not report UI when surveyed had a positive pad test.^49^ It is important to de-normalise UI among female athletes through education.^50^ A recent study surveying exercise and health professionals found that screening for pelvic floor symptoms such as UI was not common practice among these professionals and cited a lack of confidence, knowledge and training as barriers. This all highlights a need for an education programme among these elite players, as well as the coaches and health professionals involved, to increase awareness.

Whilst players spoke of managing UI using strategies, few spoke to others about their UI or sought advice from a health professional, and this corresponds with the findings of the cross-sectional study investigating UI among elite Gaelic sports athletes.^28^ Three players had sought treatment or had used PFMT to address their UI; however, these either had a health professional background or had given birth. Recent randomised controlled trials among female volleyball players^51^ and female functional fitness athletes^52^ have shown that PFMT programmes are effective in treating UI among athletes. In addition, findings of a Cochrane review reported that PFMT can cure or improve UI and is the most effective treatment of SUI,^53^ which was the type of UI most commonly experienced.

Many players were not confident in their current level of knowledge and expressed a desire for education regarding the condition and management of UI, including PFMT. This reflects the findings of previous research among elite female athletes, which suggest a need for education regarding UI and its management.^2,4,9,12 ^23^,28,54^

### Limitations

The main limitations of this research were the fact that only nine players participated in one-to-one interviews. One of the sub-themes generated included a *reticence to disclose* and talk about UI, and this may also help explain the low uptake for this study. However, despite this, the data collected were rich and new knowledge was added to the field of study.

In addition, this study involved female Gaelic sports athletes who were at least 18 years old, predominantly nulliparous, and played at an elite level. Consequently, the findings may not be generalisable to younger or club-level female Gaelic sports athletes, or to other sports. Finally, this research predominantly focused on the experience of UI with minimal information sought regarding other types of PFD among these athletes.

## Conclusions

Four key themes and nine sub-themes were generated in this qualitative study, providing in-depth knowledge regarding the players’ experience of UI. Players spoke of triggers for their UI, including high-impact activities, indicating that SUI was the most commonly experienced form of UI. All players adopted strategies to manage their UI, and few spoke about, or sought treatment for, their UI, whilst all experienced an emotional impact resulting from their UI, and many expressed an interest in learning more about the condition. Some of the practical issues, such as the availability of facilities, could easily be addressed by education and an increased awareness of those within the sporting bodies. The findings of this study indicate a need for education programmes and resources, including information about UI as well as the treatment options available. Further research should evaluate the effectiveness of such an education programme. In addition, the authors suggest that future research may also involve a randomised controlled trial to evaluate the impact of a PFMT programme on the experience of UI among these elite female Gaelic sports athletes.

## Supplemental Material

sj-docx-1-whe-10.1177_17455057251406949 – Supplemental material for Elite female Gaelic sports athletes’ experience of urinary incontinence: A qualitative study Supplemental material, sj-docx-1-whe-10.1177_17455057251406949 for Elite female Gaelic sports athletes’ experience of urinary incontinence: A qualitative study by Elizabeth Culleton-Quinn, Kari Bø, Neil Fleming, Cinny Cusack and Déirdre Daly in Women's Health

sj-docx-2-whe-10.1177_17455057251406949 – Supplemental material for Elite female Gaelic sports athletes’ experience of urinary incontinence: A qualitative study Supplemental material, sj-docx-2-whe-10.1177_17455057251406949 for Elite female Gaelic sports athletes’ experience of urinary incontinence: A qualitative study by Elizabeth Culleton-Quinn, Kari Bø, Neil Fleming, Cinny Cusack and Déirdre Daly in Women's Health

## References

[1] Haylen BT de Ridder D Freeman RM , et al. An International Urogynecological Association (IUGA)/International Continence Society (ICS) joint report on the terminology for female pelvic floor dysfunction. Int Urogynecol J 2010; 21(1): 5–26. 19937315 10.1007/s00192-009-0976-9

[2] Almousa S Bandin Van Loon A . The prevalence of urinary incontinence in nulliparous female sportswomen: a systematic review. J Sports Sci 2019; 37(14): 1663–1672. 30822258 10.1080/02640414.2019.1585312

[3] Cerruto MA Balzarro M Rubilotta E , et al. Lower urinary tract and gastrointestinal dysfunction in sportswomen: a systematic review and meta-analysis of observational studies. Minerva Urol Nefrol 2020; 72(6): 698–711. 31692306 10.23736/S0393-2249.19.03582-3

[4] de Mattos Lourenco TR Matsuoka PK Baracat EC , et al. Urinary incontinence in female athletes: a systematic review. Int Urogynecol J 2018; 29(12): 1757–1763. 29552736 10.1007/s00192-018-3629-z

[5] Dominguez-Antuña E Diz JC Suárez-Iglesias D , et al. Prevalence of urinary incontinence in female CrossFit athletes: a systematic review with meta-analysis. Int Urogynecol J 2023; 34(3): 621–634. 35635565 10.1007/s00192-022-05244-z PMC9150382

[6] Gan ZS Smith AL . Urinary incontinence in elite female athletes. Curr Urol Rep 2023; 24(2): 51–58. 36418531 10.1007/s11934-022-01133-6

[7] García-Perdomo HA Uribe Bayona AJ Zamora Segura BD . High-impact exercises associated with an increased risk of stress urinary incontinence: systematic review and meta-analysis. Curr Phys Med Rehabil Rep 2022; 10(3): 206–215.

[8] Kneißler E Zentgraf K . Prevalence of urinary incontinence in female gymnasts: a systematic review. Ger J Exerc Sport Res 2024; 55: 159–166.

[9] Pires T Pires P Moreira H , et al. Prevalence of urinary incontinence in high-impact sport athletes: a systematic review and meta-analysis. J Hum Kinet 2020; 73: 279–288. 32774559 10.2478/hukin-2020-0008 PMC7386138

[10] Rebullido TR Gómez-Tomás C Faigenbaum AD , et al. The prevalence of urinary incontinence among adolescent female athletes: a systematic review. J Funct Morphol Kinesiol 2021; 6(1): 12. 33525502 10.3390/jfmk6010012 PMC7931053

[11] Syeda F Pandit U . Urinary incontinence in female athletes: a systematic review on prevalence and physical therapy approaches. Cureus 2024; 16(7): e64544. 10.7759/cureus.64544 PMC11322629 39144856

[12] Teixeira RV Colla C Sbruzzi G , et al. Prevalence of urinary incontinence in female athletes: a systematic review with meta-analysis. Int Urogynecol J 2018; 29(12): 1717–1725. 29654349 10.1007/s00192-018-3651-1

[13] Moore IS Crossley KM Bo K , et al. Female athlete health domains: a supplement to the International Olympic Committee consensus statement on methods for recording and reporting epidemiological data on injury and illness in sport. Br J Sports Med 2023; 57(18): 1164–1174. 37349084 10.1136/bjsports-2022-106620 PMC10579182

[14] Komorowski L Chen B . Female urinary incontinence in china: experiences and perspectives. Health Care Women Int 2006; 27(2): 169–181. 16484160 10.1080/07399330500457887

[15] Lindgren A Dunberger G Enblom A . Experiences of incontinence and pelvic floor muscle training after gynaecologic cancer treatment. Support Care Cancer 2017; 25: 157–166. 27596267 10.1007/s00520-016-3394-9 PMC5127854

[16] Moossdorff-Steinhauser HFA Houkes I Berghmans BCM , et al. Experiences of peri-partum urinary incontinence from a women’s and health care perspective: a qualitative study. Matern Child Health J 2023; 27(7): 1199–1207. 36988797 10.1007/s10995-023-03631-6 PMC10232634

[17] Erkal Aksoy Y Akın B Dereli Yılmaz S . Urinary incontinence experiences of pregnant women: a qualitative study. Urologia 2020; 88(2): 140–147. 33245027 10.1177/0391560320974880

[18] Lind H Waldenström A Dunberger G , et al. Late symptoms in long-term gynaecological cancer survivors after radiation therapy: a population-based cohort study. Br J Cancer 2011; 105: 737–745. 21847122 10.1038/bjc.2011.315 PMC3171018

[19] Corrado B Giardulli B Polito F , et al. The impact of urinary incontinence on quality of life: a cross-sectional study in the metropolitan city of Naples. Geriatrics (Basel) 2020; 5(4): 96. 33233663 10.3390/geriatrics5040096 PMC7709681

[20] Toye F Barker KL . A meta-ethnography to understand the experience of living with urinary incontinence: ‘is it just part and parcel of life?’. BMC Urol 2020; 20(1): 1. 31941470 10.1186/s12894-019-0555-4 PMC6964106

[21] Mendes A Hoga L Gonçalves B , et al. Adult women’s experiences of urinary incontinence: a systematic review of qualitative evidence. JBI Database System Rev Implement Rep 2017; 15(5): 1350–1408. 10.11124/JBISRIR-2017-003389 28498174

[22] Dakic JG Hay-Smith J Cook J , et al. Effect of pelvic floor symptoms on women’s participation in exercise: a mixed-methods systematic review with meta-analysis. J Orthop Sports Phys Ther 2021; 51(7): 345–361. 33971737 10.2519/jospt.2021.10200

[23] Culleton-Quinn E Bø K Fleming N , et al. Elite female athletes’ experiences of symptoms of pelvic floor dysfunction: a systematic review. Int Urogynecol J 2022; 33(10): 2681–2711. 36040507 10.1007/s00192-022-05302-6 PMC9477953

[24] Dakic JG Hay-Smith J Lin K-Y , et al. Experience of playing sport or exercising for women with pelvic floor symptoms: a qualitative study. Sports Med Open 2023; 9(1): 25. 37097457 10.1186/s40798-023-00565-9 PMC10127961

[25] Jácome C Oliveira D Marques A , et al. Prevalence and impact of urinary incontinence among female athletes. Int J Gynecol Obstet 2011; 114(1): 60–63. 10.1016/j.ijgo.2011.02.004 21571270

[26] Camogie Association. Official Rules, https://www.camogie.ie/wp-content/uploads/2021/05/Part-2-Official-Rules-2021-A4-Final.pdf (2023).

[27] LGFA. Playing Rules 2021, https://ladiesgaelic.ie/wp-content/uploads/2018/03/Playing-Rules-2021-1.pdf (2021).

[28] Culleton-Quinn E Bø K Fleming N , et al. Prevalence and experience of urinary incontinence among elite female gaelic sports athletes. Int Urogynecol J 2024; 35(12): 2357–2365. 39150568 10.1007/s00192-024-05893-2 PMC11732913

[29] Sandelowski M . Whatever happened to qualitative description? Res Nurs Health 2000; 23(4): 334–340. 10940958 10.1002/1098-240x(200008)23:4<334::aid-nur9>3.0.co;2-g

[30] Morgan D . Integrating qualitative and quantitative methods: a pragmatic approach. SAGE Publications, Inc., 2014.

[31] Kaushik V Walsh CA . Pragmatism as a research paradigm and its implications for social work research. Soc Sci 2019; 8(9): 255.

[32] Tong A Sainsbury P Craig J . Consolidated criteria for reporting qualitative research (COREQ): a 32-item checklist for interviews and focus groups. Int J Qual Health Care 2007; 19(6): 349–357. 17872937 10.1093/intqhc/mzm042

[33] Braun V Clarke V . Thematic analysis: a practical guide. SAGE Publications, 2021.

[34] Guba E Lincoln Y . Competing paradigms in qualitative research. In: Denzin NK and Lincoln (eds) Handbook of qualitative research. Sage Publications, Inc., 1994, pp.105–117.

[35] Carcary M . The research audit trial–enhancing trustworthiness in qualitative inquiry. The Electron J Bus Res Methods Vol 2009; 7: 11–24.

[36] Birt L Scott S Cavers D , et al. Member checking: a tool to enhance trustworthiness or merely a nod to validation? Qual Health Res 2016; 26(13): 1802–1811. 27340178 10.1177/1049732316654870

[37] Hennink MM Kaiser BN Marconi VC . Code saturation versus meaning saturation: how many interviews are enough? Qual Health Res 2016; 27(4): 591–608. 27670770 10.1177/1049732316665344 PMC9359070

[38] Carvalhais A Natal Jorge R Bø K . Performing high-level sport is strongly associated with urinary incontinence in elite athletes: a comparative study of 372 elite female athletes and 372 controls. Br J Sports Med 2018; 52(24): 1586–1590. 28642223 10.1136/bjsports-2017-097587

[39] Johnston CL Negus MF Rossiter MA , et al. A national survey of urinary incontinence in professional Team England female athletes. Eur J Obstet Gynecol Reprod Biol 2023; 282: 12–16. 36608453 10.1016/j.ejogrb.2022.12.031

[40] Simeone C Moroni A Pettenò A , et al. Occurrence rates and predictors of lower urinary tract symptoms and incontinence in female athletes. Urologia 2010; 77(2): 139–146. 20890872

[41] Rodríguez-López ES Calvo-Moreno SO Basas-García Á , et al. Prevalence of urinary incontinence among elite athletes of both sexes. J Sci Med Sport 2021; 24(4): 338–344. 33041208 10.1016/j.jsams.2020.09.017

[42] Thomaz RP Colla C Darski C , et al. Influence of pelvic floor muscle fatigue on stress urinary incontinence: a systematic review. Int Urogynecol J 2018; 29(2): 197–204. 29264615 10.1007/s00192-017-3538-6

[43] Hextall A Bidmead J Cardozo L , et al. The impact of the menstrual cycle on urinary symptoms and the results of urodynamic investigation. BJOG 2001; 108(11): 1193–1196. 11762662 10.1111/j.1471-0528.2003.00280.x

[44] Hvidman L Foldspang A Mommsen S , et al. Does urinary incontinence occurrence depend on the menstrual cycle phase? Acta Obste Gynecol Scand 2002; 81(4): 347–350. 10.1034/j.1600-0412.2002.810413.x 11952467

[45] Hannigan M . Skorts: what are they and why are we suddenly talking about them? Many camogie players find the mandated skirt-short hybrids restricting, but it seems they’ll be stuck wearing them until 2027 at least. The Irish Times, https://www.irishtimes.com/life-style/2024/04/12/give-me-a-crash-course-in-the-skorts-controversy/ (2024).

[46] Cormican E . Voting to maintain discomfort for camogie players. Really? The Irish Examiner, https://www.irishexaminer.com/sport/gaa/arid-41369341.html (2024).

[47] Deshayes TA Jeker D Goulet EDB . Impact of pre-exercise hypohydration on aerobic exercise performance, peak oxygen consumption and oxygen consumption at lactate threshold: a systematic review with meta-analysis. Sports Med 2020; 50(3): 581–596. 31728846 10.1007/s40279-019-01223-5

[48] Cygańska AK Sobieska D Truszczyńska-Baszak A , et al. Women’s awareness of physiotherapeutic methods in the treatment of urinary incontinence. Adv Rehabil 2018; 32(4): 21–27.

[49] Dos Santos KM Da Roza T Tonon da Luz SC , et al. Quantification of urinary loss in nulliparous athletes during 1 hour of sports training. PM R 2019; 11(5): 495–502. 30179664 10.1016/j.pmrj.2018.08.383

[50] Donnelly GM Moore IS . Sports medicine and the pelvic floor. Curr Sports Med Rep 2023; 22(3): 82–90. 36866951 10.1249/JSR.0000000000001045

[51] Ferreira S Ferreira M Carvalhais A , et al. Reeducation of pelvic floor muscles in volleyball athletes. Rev Assoc Med Bras 2014; 60(5): 428–433.

[52] Skaug KL Engh ME Bø K . Pelvic floor muscle training in female functional fitness exercisers: an assessor-blinded randomised controlled trial. Br J Sports Med 2024; 58(9): 486–493. 38413133 10.1136/bjsports-2023-107365 PMC11103308

[53] Dumoulin C Cacciari LP Hay-Smith EJC . Pelvic floor muscle training versus no treatment, or inactive control treatments, for urinary incontinence in women. Cochrane Database Syst Rev 2018; 2018(10): CD005654. 10.1002/14651858.CD005654.pub4 PMC6516955 30288727

[54] Mahoney K Heidel RE Olewinski L . Prevalence and normalization of stress urinary incontinence in female strength athletes. J Strength Cond Res 2023; 37(9): 1877–1881. 36930880 10.1519/JSC.0000000000004461 PMC10448802

