# Supplementary material for: Elite female Gaelic sports athletes’ experience of urinary incontinence: A qualitative study
Source: Womens Health (Lond). 2025 Dec 24;21:17455057251406949. doi: 10.1177/17455057251406949 (PMC12744028; doi:10.1177/17455057251406949)
Supplement: sj-docx-2-whe-10.1177_17455057251406949 – Supplemental material for Elite female Gaelic sports athletes’ experience of urinary incontinence: A qualitative study [file sj-docx-2-whe-10.1177_17455057251406949.docx]

**Appendix 2 Semi-structured Interview Guide**

- Do you leak in training- if so, when? Prompt? Start? End?
- Is there anything that you can think about that triggers a leak in training? Prompt
- Do you leak in matches- if so, when? Start? End?
- Is there anything that you can think about that triggers a leak in matches? Prompt
- Do you leak outside of sport- if so, when? Do you leak when you cough or sneeze or move suddenly? Do you ever get a sudden urge to go to the toilet to pass water? Yes- would you ever start to leak
- Can you think of any other triggers for your UI in 1.sport/2.daily life? (a/a)
- Is it (UI) different at any particular time –Prompt notice anything around periods or during your menstrual cycle?
- Do you do anything in particular to help manage your leaking in 1.sport/2.daily life (a/a)? Strategies – prompt…pre-void, fluid restriction, wear pads (what type), clothes
- Do you limit your sporting activity due to leakage? If so, how do you limit?
- Does leaking in sport affect you? If so, how?
- Have you spoken about it with anyone else? Yes-Who. No -Why do you think that is? Do you think that many players would talk about it
- What facilities are available during sport (matches and training)? Does this have any effect on your UI?
- Have you ever looked for treatment for your leakage? Yes- from who? No- Did you know it was treatable?
- Have you ever heard about UI/BL in sport before?
- Do you know where PFM are?
- Have you heard about PFExs/PFMT? How/where did you learn about them?
- Do you feel confident about doing PFMT for e.g. pelvic floor exercises? Do you do any of them? if so, how often? If not any particular reason? Where did you get your information re PFMT?
- Do you have any other symptoms in the pelvic floor? Bowel symptoms. Pain in pelvic floor- Difficulties in that area that you are happy to talk about.
- Is there anything else you would like to mention about your UI?
